# Patient with Vulnerable Coronary Plaque and Treatment with Evolocumab: A Clinical Case

**DOI:** 10.3390/jcm14041257

**Published:** 2025-02-14

**Authors:** Lucio Addeo, Pasquale Guarini, Pasquale Campana, Luigi Argenziano, Stefano Nardi, Carlo Tedeschi, Alessandra Scatteia, Mattia Silvestre, Antonio Rapacciuolo, Giovanni Esposito, Salvatore Giordano, Laura Adelaide Dalla Vecchia, Francesco Donatelli

**Affiliations:** 1 Department of Advanced Biomedical Sciences, University of Naples Federico II, Via Sergio Pansini, 80131 Naples, Italy; 2 U.O Cardiologia, Clinica Sanatrix, Centro Studi SICOA, 80127 Naples, Italy; 3 Dipartimento di Cardiologia, Pineta Grande Hospital, Castel Volturno, 81030 Caserta, Italy; 4 IRCCS SYNLAB SDN, Via Emanuele Gianturco 113, 80143 Naples, Italy; 5 Unità di Imaging Cardiovascolare Avanzato, Clinica Villa dei Fiori, Acerra, 80011 Naples, Italy; 6 Unità di Radiologia, Pineta Grande Hospital, Castel Volturno, 81030 Caserta, Italy; 7 Department of Medical and Surgical Sciences, Division of Cardiology, ‘Magna Graecia’ University, 88100 Catanzaro, Italy; 8 Department of Cardiology, IRCCS Istituti Clinici Scientifici Maugeri, 20138 Milan, Italy; 9 Department of Clinical and Community Sciences, University of Milan, 20122 Milan, Italy

**Keywords:** plaque stabilization, PCSK-9 inhibitors, coronary CT angiography

## Abstract

**Background/Objectives:** Vulnerable coronary plaques are strongly associated with acute coronary events, posing significant therapeutic challenges despite statin therapy. This case report evaluates the impact of Evolocumab, a PCSK-9 inhibitor, on stabilizing high-risk plaques and promoting phenotypic transformation, assessed through coronary CT angiography (CCTA). **Methods:** A 50-year-old male with chronic coronary syndrome and a history of myocardial infarction underwent a CCTA, revealing a high-risk plaque (approximately 50%) in the proximal LAD. Despite achieving LDL-C targets with statin therapy, the plaque showed vulnerability features. Evolocumab (140 mg subcutaneously every two weeks) was added to therapy, combined with dietary counseling and dual antiplatelet therapy. **Results:** A follow-up CCTA at 24 months demonstrated significant reductions in plaque volume and positive remodeling, with a transformation from a mixed phenotype to a predominantly calcified plaque. LDL-C levels decreased from 71 mg/dL to 18 mg/dL. The patient remained asymptomatic, with no cardiovascular events reported during the follow-up. **Conclusions:** This case highlights the role of PCSK-9 inhibitors in stabilizing high-risk plaques, achieving structural changes that promote stability beyond LDL-C reduction. Advanced imaging techniques such as CCTA proved essential for risk stratification and monitoring therapy efficacy. Evolocumab offers a promising adjunctive treatment for high-risk patients unsuitable for elective revascularization, potentially redefining the standard of care for plaque stabilization in this setting.

## 1. Introduction

Coronary artery disease (CAD) remains a leading cause of morbidity and mortality worldwide, also due to acute events triggered by rupture-prone, vulnerable plaques which contribute to the residual inflammatory risk [1]. Characterized by thin fibrous caps, large lipid cores, and positive remodeling, these plaques pose a significant challenge despite advances in medical and interventional therapies [2]. Non-invasive imaging, particularly coronary CT angiography (CCTA), has transformed plaque detection, enabling early risk stratification and targeted interventions [3]. However, some patients fail to achieve optimal LDL-C levels with statin therapy alone, leaving them at residual cardiovascular risk due to persistent plaque vulnerability [4]. This case report describes a middle-aged patient who underwent successful PCI of the Circumflex artery (Cx) but had a residual high-risk plaque in the left anterior descending artery (LAD), identified via a CCTA. Despite being unsuitable for revascularization, the lesion could benefit from intensive LDL-C reduction to achieve significant morphological regression, as reported by a number of studies [5,6]. The results of such an approach may highlight and confirm the pivotal role of adjunct lipid-lowering strategies in stabilizing high-risk plaques in a real-world case. Additionally, integrating a CCTA with functional tests could enhance risk assessment by providing deeper insights into plaque vulnerability and coronary flow impairment, further informing treatment decisions for complex CAD cases.

## 2. Case Presentation

**Figure 1:**
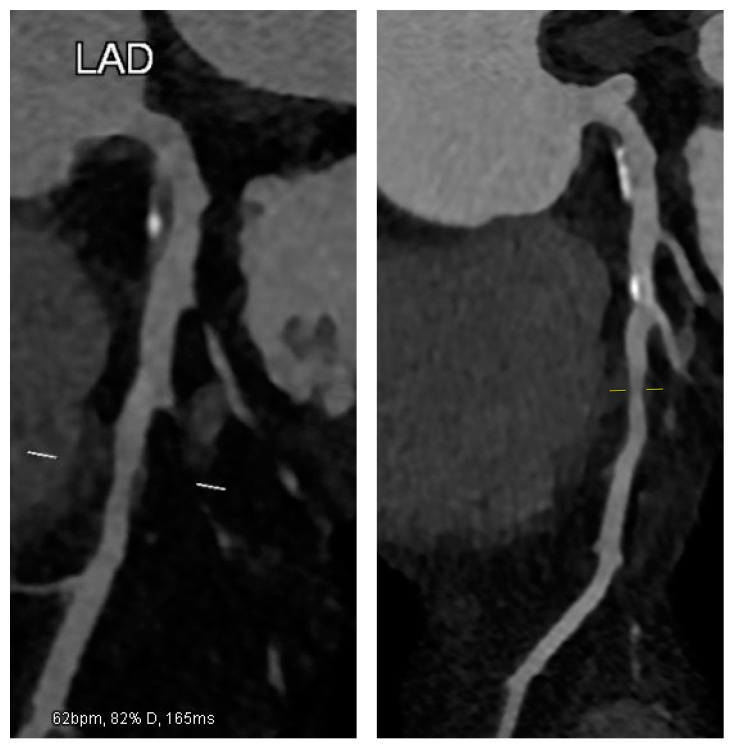
CCTA reveals a high-risk mixed plaque in the proximal LAD with a predominantly non-calcified component (**left**). After Evolocumab therapy, the plaque transforms into a more calcified, stable phenotype with reduced volume (**right**).

**Figure 2:**
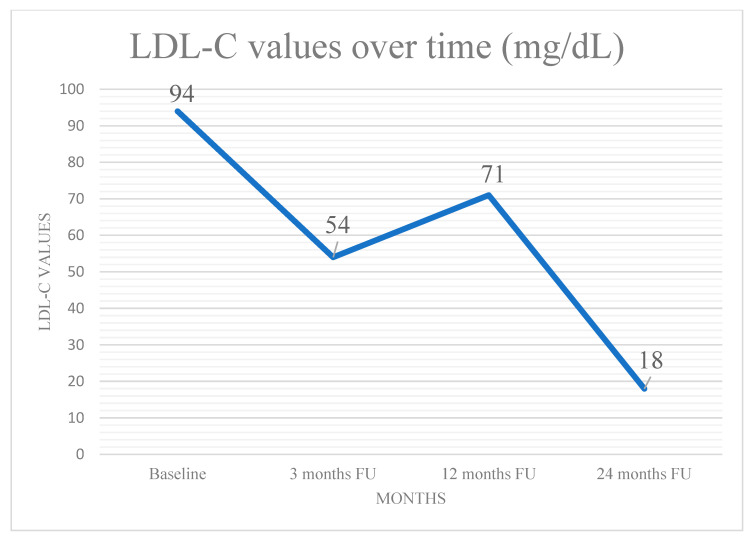
LDL-C values post-PCI and during follow-up.

A 50-year-old male with chronic coronary syndrome presented after a recent inferolateral myocardial infarction (MI). His history included a prior posterolateral STEMI, treated with PCI and stenting of a culprit lesion in the Cx. A non-significant yet non-negligible (approximately 50%) ostial LAD stenosis was observed but not further evaluated with IVUS or OCT. His cardiovascular risk factors included mixed dyslipidemia (pre-admission LDL-C: 123 mg/dL), hypertension, overweight status, and a history of smoking, which he quit post-MI. At baseline (within 30 days), he was prescribed Bisoprolol, Ramipril, Ticagrelor, Aspirin, and Atorvastatin 80 mg daily. Despite occasional non-adherence, he reported exertional dyspnea without chest pain. Due to an LDL-C of 94 mg/dL, therapy was switched to Rosuvastatin plus Ezetimibe, with adherence counseling. At three months, echocardiography showed preserved LV function with localized hypokinesia of the posterolateral wall. The patient reported good compliance, achieved an LDL-C of 54 mg/dL, and remained asymptomatic. A follow-up was planned within a year. At 12 months, LDL-C rose modestly to 71 mg/dL, slightly above target, attributed to mild weight gain from a suboptimal diet despite reported adherence. To refine risk stratification, CCTA was selected as the imaging modality of choice, aligned with current guidelines [7,8]. An ECG-gated CCTA was performed using a single-source 128-slice CT scanner (Somatom go. Top 128, Siemens, Germany). The patient was hemodynamically stable, with a heart rate < 60 bpm after infusion of Metoprolol. Nitroglycerin was administered for optimal coronary visualization. Imaging was acquired in a single end-inspiratory breath-hold, covering the area from the carina to the diaphragm. A total of 70 mL of high-concentration iodinated contrast (370 mgI/mL) was injected at 5 mL/s, followed by 40 mL of saline at the same rate. Retrospectively ECG-gated imaging was triggered by ascending aortic opacification (>150 HU). Axial images were reconstructed at a 0.4 mm slice thickness using iterative methods. High-risk plaque (HRP) parameters included low-attenuation plaque (LAP), spotty calcification (SC), and positive remodeling (PR). LAP was defined as any pixel with HU ≤ 30 within the lesion. SC was a small (<3 mm), dense (>130 HU) plaque component surrounded by non-calcified tissue. PR was defined by a remodeling index ≥ 1.1 (maximal lesion vessel area/proximal reference vessel area). A CT scan revealed a high-risk plaque in the proximal LAD, characterized by a predominantly non-calcified component, low-attenuation, positive remodeling, and spotty calcifications, all indicative of plaque vulnerability (Figure 1). In response, the therapeutic strategy was adjusted: Ticagrelor was reduced (not discontinued), and Evolocumab (140 mg SC every two weeks) was introduced. The patient was counseled on a lipid-lowering diet and regular aerobic exercise. At 24 months, the patient remained asymptomatic, with stable weight and good adherence to therapy. Echocardiography showed unchanged LV function. LDL-C was significantly reduced to 18 mg/dL (Figure 2). A repeat CCTA using the same protocol, operator, and scanner revealed a transformation in plaque phenotype, with the high-risk plaque evolving into a predominantly calcified form and an increased vessel lumen, indicative of greater stability and lower rupture risk [9,10]. Plaque volume, stenosis, and positive remodeling were substantially reduced (Figure 1). Based on these findings, the therapeutic regimen was maintained, except for Ticagrelor discontinuation. A follow-up was scheduled within one year or earlier if symptoms developed.

## 3. Discussion

This case illustrates an interesting strategy for managing residual cardiovascular risk post-MI by incorporating recent advancements into clinical practice. At 12 months post-MI, a CCTA identified a high-risk proximal LAD plaque with positive remodeling and a low-attenuation non-calcified component, resulting crucial for risk stratification and therapeutic planning [11,12]. Libby and Pasterkamp emphasized the need to consider systemic and inflammatory factors beyond structural features [4], aligning with our case, which highlights the importance of addressing both plaque morphology and systemic risk factors. A follow-up CCTA showed a significant transformation, with the plaque becoming predominantly calcified, alongside reductions in volume and positive remodeling. These findings suggest that PCSK9 inhibition played a key role in plaque stabilization [9,10]. Evolocumab, known for its potent LDL-C-lowering effects, also modifies plaque composition, enhancing stability. This aligns with evidence from Nicholls et al., who demonstrated its ability to reduce plaque burden and improve stability, even in patients with well-controlled LDL-C on statins [6]. The PROSPECT study underscored the importance of addressing non-culprit lesions, reporting similar event rates for culprit and non-culprit plaques over three years (12.9% vs. 11.6%) [13]. This reinforces the need for comprehensive post-PCI evaluation, as non-obstructive plaques contribute to future events. CCTA has proven to be indispensable in detecting high-risk plaques and guiding management [3]. PCSK9 inhibitors represent a major advancement in stabilizing such plaques, promoting calcification, and addressing cardiovascular risk factors beyond LDL-C reduction [14]. Emerging evidence suggests PCSK9 influences LDLR expression on macrophages [15], modulating lipid uptake and contributing to plaque formation. Additionally, PCSK9 may activate pathways (Syk, PKCδ, NF-κB) that drive lipid accumulation and lesion development [16], potentially explaining its direct impact on plaque progression. The GLAGOV trial demonstrated that PCSK9 inhibitors not only lower LDL-C but also induce favorable changes in plaque morphology [5], consistently with the PROVE-IT TIMI 22 trial, which confirmed the benefits of intensive lipid-lowering in stabilizing plaques and reducing recurrent events [14]. Current ACC/AHA and ESC/EAS guidelines support PCSK9 inhibitors in high-risk populations when LDL-C targets are unmet or further plaque stabilization is needed [17,18]. Expanding their use in patients with high-risk plaque features, even on optimal statin therapy, aligns with existing evidence [6,19], addressing residual cardiovascular risk. However, whether aggressive LDL-C lowering and plaque calcification translate into improved long-term outcomes—considering factors like age, sex differences, and comparisons with preventive PCI—remains uncertain [20,21,22]. Additionally, macrocalcification may reduce the success rate of future PCI if needed [23]. While the plaque-stabilizing effects of Evolocumab are well established and regression has been demonstrated through invasive imaging, this is the first reported case documenting such significant regression (~50%) of an ostial LAD plaque using non-invasive imaging (CCTA) and PCSK9 inhibition. This reinforces CCTA’s role in tracking plaque evolution and guiding therapy. An integrated approach, including functional testing, may refine risk stratification and identify patients who benefit most from medical therapy. Future research should explore the long-term impact of PCSK9 inhibitors across subgroups and their potential synergies with other lipid-lowering agents to optimize outcomes.

## 4. Conclusions

This report emphasizes the role of CCTA as an advanced imaging technique in guiding therapy and risk stratification for patients not eligible for elective revascularization, thus allowing the selection of patients who can benefit more from intensive lipid-lowering therapy, and highlights PCSK9 inhibitors’ potential in plaque stabilization and cardiovascular residual risk reduction. Future research should validate these findings in larger cohorts, assess long-term PCSK9 inhibitor effects, and explore biomarkers for monitoring plaque transformation. Investigating genetic and molecular determinants of plaque vulnerability may further refine therapy. Randomized trials combining PCSK9 inhibitors with statins or ezetimibe could reveal synergistic benefits, particularly for high-risk residual plaques unsuitable for PCI. Additional studies should evaluate cost-effectiveness, safety, and long-term adherence. Integrating advanced imaging into routine workflows may improve risk assessment, optimize treatment, and enhance outcomes, reshaping CAD management for patients ineligible for invasive interventions.

## 5. Limitations

Growing evidence suggests that PCSK9 inhibitors enhance plaque stability by increasing the fibrous cap thickness and lumen area, though the molecular mechanisms still need to be explored. While this case report does not address this gap, it underscores the need for further research to guide clinical management. Despite its limitations, the morphological changes observed in a typical CAD patient highlight the importance of identifying residual risk and personalizing treatment. These findings should be validated in larger cohorts, preferably through randomized, multicenter trials, to provide more robust evidence and stronger insights in this setting.

## Data Availability

The original contributions presented in this study are included in the article. Further inquiries can be directed to the corresponding author (addeolucio@gmail.com).

## Abbreviations

The following abbreviations are used in this manuscript:

CAD	Coronary artery disease
CCTA	Coronary CT angiography
Cx	Circumflex artery
LAD	Left anterior descending artery
MI	Myocardial infarction
PCI	Percutaneous coronary intervention
